# TNFα Inhibitors Versus Newer Therapies in Spondyloarthritis: Where do we Stand Today?

**DOI:** 10.31138/mjr.040224.tvn

**Published:** 2024-12-31

**Authors:** Alexandros A. Drosos, Eleftherios Pelechas, Aliki I. Venetsanopoulou, Paraskevi V. Voulgari

**Affiliations:** Rheumatology Clinic, Department of Internal Medicine, Medical School, University of Ioannina, Ioannina, Greece

**Keywords:** SpA, ax-SpA, cytokines, TNFα inhibitors, IL-17 inhibitors, IL-12/IL-23 inhibitors, JAK inhibitors, adverse drug reactions

## Abstract

The spondyloarthritides (SpA) are a group of chronic inflammatory diseases that affect the axial skeleton (ax-SpA), peripheral joints and entheses (p-SpA) and are expressed with several clinical phenotypes such as psoriasis, psoriatic arthritis (PsA), inflammatory bowel disease (IBD), and uveitis. The pathogenesis of SpA involves the pivotal role of tumour necrosis factor alpha (TNFα) and the interleukins (IL) IL-17/IL-23. Their distribution and hierarchy in the affected organs and tissues is differently expressed in SpA. TNFα is expressed in all tissues and organs, while IL-17 and IL-12/IL-23 is lacking from the gut and the axial skeleton respectively. This knowledge is a dilemma for physicians when they must choose a biological therapy. Nowadays, the armamentarium of SpA treatment has been expanded comprising biological therapies such as TNFα inhibitors (TNFαi), IL-17 inhibitors (IL-17i), IL-12/IL-23 inhibitors (IL-12/IL-23i), as well as the Janus Kinase inhibitors (JAKi). Several studies have shown that IL-12/IL-23i are very effective to treat psoriasis, PsA and IBD, but are ineffective in treating ax-SpA. IL-17i are very effective in patients with ax-SpA, psoriasis and PsA, but seem ineffective in IBD. Finally, TNFαi have shown to be effective in all SpA phenotypes with an acceptable toxicity profile. On the other hand, JAKi are also effective in almost all SpA phenotypes, but caution is required for elderly patients who may develop Herpes-Zoster infection, thromboembolic events and malignancies. However, the treatment of SpA is individualised according to the clinical phenotype and after shared decision between patients and physicians.

## INTRODUCTION

The spondylarthritides (SpA) are a group of chronic inflammatory diseases that affect the axial skeleton (ax-SpA) mostly the sacroiliac joints (SIJ) and spine and/or the peripheral joints (p-SPA) and entheses. It affects young adults of both sexes and starts with low back pain (LBP) and morning stiffness. The prototype of ax-SpA is ankylosing spondylitis (AS) with evidence of structural radiological damage of the SIJ (sacroiliitis) (r-ax-SpA). However, there are patients with chronic LBP, without evidence of sacroiliitis on conventional radiographs (CR), named nr-ax-SpA. It is believed that nr-ax-SpA represents an early stage of SpA. The extraskeletal manifestations (ESM) include psoriasis, inflammatory bowel disease (IBD) and uveitis.^1–3^ The diagnosis of SpA relies on the recognition of the disease’s clinical symptoms and signs, as well as imaging of the SIJ using CR and/or magnetic resonance imaging (MRI).^4^ In the last decades, a delay of SpA diagnosis has been noted that can last up to 7 years, due to different classification criteria used by investigators. However, with the introduction of the Assessment of Spondyloarthritis International Society (ASAS) classification criteria and the use of MRI of the SIJ, the recognition of SpA and its phenotypes in early disease stage is now possible, because the ASAS criteria include the whole spectrum of SpA, as well as ESM.^5,6^

## PATHOGENESIS

The hallmark of the pathologic events in SpA are entheseal and subchondral bone involvement. Entheseal pathophysiology occurs in predisposed individuals with a specific genetic background. The human leucocyte antigen (HLA) B-27 along with the endoplasmatic reticulum aminopeptidase-1 (ERAP-1) and the interleukin (IL)-23 receptor (IL-23R) variants are some of the genetic factors.^7,8^ Entheses are load-bearing structures receiving mechanical stress. Furthermore, skin damage induced by psoriasis and intestinal mucosa by IBD, facilitate the exposure to pathogens. Thus, in susceptible individuals continuous mechanical stress and infections may cause microdamage to entheses, leading to activation of the innate immune cells [innate lymphoid cells-3 (ILC-3), γδ-Tcells, mucosa associated invariant T-cells (MAIT), macrophages] as well as the activation of the adaptive immune cells (Th-1 and Th-2).

**Figure 1.**
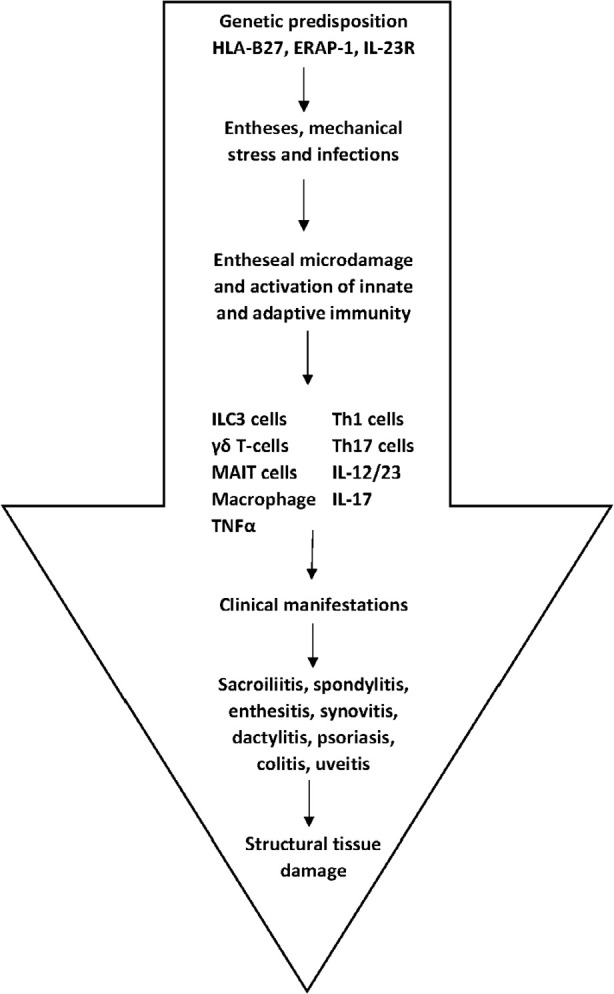
Schematic representation of SpA pathophysiology. HLA-B27: Human Leucocyte Antigen B27; ERAP-1: Endoplasmatic Reticulum Aminopeptidase 1; IL-23R: Interleukin 23 Receptor; ILC3: Innate Lymphoid type 3 Cells; MAIT: mucosa-associated invariant T-cells.

As a result, several cytokines are produced [tumour necrosis factor alpha (TNFα), IL-12/IL-23 and IL-17] which play a central role in disease pathogenesis. These cytokines are responsible for the clinical manifestations, disease progression and tissue damage (Figure 1).^9–11^ However, not all cytokines mentioned above are expressed equally at the specific sites of inflammation. They are expressed with a different hierarchy and distribution. Thus, in the axial skeleton there is an IL-17 and TNFα predominance, while in the gut TNFα and IL-12/IL-23 with a lack of IL-17. On the other hand, in the joints, entheses and skin, all cytokines are expressed but with different distribution and hierarchy.^12,13^ The aforementioned differences are very important for physicians when choosing biological agents to treat SpA patients (**Table 1**).

**Table 1.** Cytokine expression, its hierarchy in SpA phenotypes, and biological treatment.

**Tissue/organ**	**Axial skeleton**	**Peripheral joint**	**Enthesis**	**Skin**	**Gut**	**Eye**
**Cytokine expression**	IL-17, TNFα	TNFα, IL-17, IL-12/23	IL-17, IL-12/23, TNFα	IL-17, IL-12/23, TNFα	TNFα, IL-12/23	TNFα
**Disease phenotype**	axSpA	Synovitis, dactylitis	Enthesitis	Psoriasis	Colitis	Uveitis
**Biological therapy**	IL-17i, TNFαi, JAKi	TNFαi, IL-17i, IL12/IL-23i, JAKi	IL-17i, IL12/IL-23i, TNFαi, JAKi	IL-17i, IL-12/IL-23i, TNFαi, JAKi	TNFαi, IL-12/IL-23i, JAKi	TNFαi

TNFαi: TNFα inhibitors; IL-17i: IL-17 inhibitors; IL-12/IL-23i: IL-12/IL-23 inhibitors; JAKi: JAK inhibitors.

## TREATMENT

TNFα was the first cytokine target in the treatment of inflammatory arthritides (IA).^14^ TNFα inhibitors (TNFαi) were the first biologic (b) disease modifying anti-rheumatic drugs (DMARDs) and signalled a new era in the management of IA. Etanercept (ETN) was the first TNFαi approved for the treatment of ax-SpA.^15^ Since then, several TNFαi have been added in the therapeutic armamentarium of SpA such as infliximab (IFX), adalimumab (ADA), golimumab (GOL), certolizumab (CTZ), and their biosimilars.^16–18^ Over the years, more cytokine inhibitors have been approved. IL-17 inhibitors (IL-17i) [secukinumab (SEC), ixekizumab (IXE)], followed by IL-12/IL-23 inhibitors [ustekinumab (UST – IL 12/23i) guselkumab (GUS), and risankizumab (RSK) (IL-23i). Lastly, targeted synthetic (ts) DMARDs such as Janus Kinase (JAK) inhibitors (JAKi) [upadacitinib (UPA), tofacitinib (TOFA)] have been approved for the treatment of SpA.^18–20^ The use of bDMARDs and tsDMARDs has been incorporated in the ASAS/European League Against Rheumatism (EULAR) recommendations for the treatment of SpA. Indeed, following the ASAS/EULAR recommendations and treatment strategies, clinical remission and low disease activity (LDA) are now a feasible option.^21^ However, bDMARDs and tsDMARDs present somewhat divergent effectiveness regarding the spectrum of SpA phenotype (**Table 1**). It seems that TNFαi and IL-17i as well as JAKi have demonstrated quite similar efficacy and safety, with some differences, while the IL-12/IL-23i have no effect on ax-SpA. On the other hand, not all TNFαi demonstrated the same effectiveness in all SpA phenotypes. Thus, ETN is effective to treat ax-SpA, but it is not effective to treat colitis or uveitis.^21^ On the other hand, the effect of IL-17i in uveitis is not well known yet; however, IL-17i may have an opposite effect on colitis causing in some cases disease exacerbation. Treatment is based on a shared decision between patients and physicians following the treat-to-target approach according to the ASAS/EULAR recommendations,^21^ and the Group for Research and Assessment of Psoriasis and Psoriatic Arthritis (GRAPPA) guidelines.^22^ The use of bDMARDs is indicated as a second-line treatment for SpA, when at least two nonsteroidal anti-inflammatory drugs (NSAIDs) used within 4 weeks have failed. In addition, SpA patients must have elevated C-reactive protein (CRP) in their serum, or imaging evidence of sacroiliitis on CR or MRI.^18,21^

### TNFα inhibitors

TNFαi were the first agents approved for the treatment of AS and their use transformed the clinical and laboratory outcome of AS.^15–17^ TNFαi structure differs regarding the way of action, but overall, all TNFαi are effective in reducing signs and symptoms and improved patients’ physical function and quality of life. Several randomised and observational clinical studies have demonstrated TNFαi’s high efficacy in AS patients who have failed in NSAIDs therapy.^15–17,21^ Furthermore, observational studies have shown a high retention rate and sustained clinical improvement over time in SpA patients.^23–25^ Male sex, non-smokers, early treatment, high CRP and MRI lesions of SIJ are associated with a better outcome.^26^ In this setting of patients with persistent clinical response TNFαi discontinuation has not been proved to be successful, since almost all patients with AS have experienced a disease flare after several months of TNFαi discontinuation.^18^ On the contrary, TNFαi dose reduction or increased interval dose administration were not inferior to standard dose treatment.^18^ However, a systematic review and meta-analysis showed that patients with ax-SpA achieved little clinical benefit from TNFαi reduction.^27^ Regarding structural damage, clinical trials initially showed negative results when TNFαi were administered for up to 2 years. However, recent studies demonstrated that TNFαi may inhibit structural damage progression^28,29^ and showed also an inhibition of osteoblast activity.^30^ These beneficial effects on radiographic progression in ax-SpA are very encouraging, since these patients had bad quality of life and poor prognosis in the past. Indeed, a recent study in AS patients treated with TNFαi showed that these patients had no excess mortality as compared to the control group.^31^ The use of TNFαi in SpA have showed also beneficial effects in patient who had a concomitant ESM like psoriasis, IBD or uveitis. Indeed, all TNFαi have demonstrated very good clinical response in patients with psoriasis. As regards IBD, TNFα monoclonal antibodies (ADA, IFX, CTZ, GOL) have showed excellent results,^32–34^ while a good clinical efficacy was noted with the use of TNFαi monoclonal antibodies in SpA patients with uveitis.^35,36^ As far as TNFαi safety is concerned, there is a wide spectrum of possible adverse events (AEs) such as viral, common and opportunistic infections.^1–3^ Other reported AEs comprise the paradoxical autoimmune skin reactions development such as psoriasis, granuloma annulare, erythematous skin lesions mimicking lupus and several others.^37,38^ Neurological manifestations have also been reported,^39,40^ along with hepatic and haematological abnormalities. Overall, TNFαi have a quite good safety profile and AEs can be prevented via baseline screening for occult infections, as well as with close follow-up and monitoring. Still, there are concerns about the immunogenicity induced during TNFαi treatment.

Finally, TNFαi biosimilars have demonstrated similar efficacy and safety in AS patients compared to their originators. Indeed, studies have showed that switching from originator IFX to its biosimilar had no differences in the means of clinical efficacy and AEs in AS patients.^41,42^

### IL-17 Inhibitors

The IL-17 family comprises 6 known cytokines IL-17A -IL-17F with the most important being IL-17A and IL-17F. They are capable of forming heterodimers activating their receptor IL-17RA and IL-17RF respectively.^43^ Several cells are capable to produce IL-17 except for CD4 T-cells and CD8 T-cells. Other cells include the ILC-3, γδT-cells, MAIT, neutrophils and others. However, most IL-17 is produced by the Th17 cells^44^ which in turn stimulate the production of TNFα, IL-23, IL-6 by synovial fibroblasts, macrophage and monocytes, leading to a positive feedback loop for Th17 cells differentiation.^45^ Two IL-17i have been licensed SEC and IXE, while bimekizumab, an IL-17A and IL-17F inhibitor, has received authorisation.^20^ Several studies investigated the efficacy of IL-17i in patients with r-ax-SpA and nr-ax-SpA. Indeed, SEC showed great improvement compared to placebo in two, phase III clinical trials. In addition, its clinical response was higher compared to placebo in patients with SpA who were TNFαi naive and in patients who had an inadequate response to TNFαi.^46–49^ IXE has been also studied in ax-SpA patients. One study included TNFαi naïve patients, while a second assessed patients on TNFαi with inadequate response. It has been shown that IXE was superior to placebo. Similar results have been shown in another trial in patients with nr-ax-SpA who were TNFαi naïve.^50–52^ IL-17i showed also, similar effects on disease control in patients with psoriasis, and psoriatic arthritis (PsA). In some cases, IL-17i showed superior efficacy in psoriatic patients compared to TNFαi. Similarly, PsA patients with axial involvement show beneficial effects using IL-17i versus non-biological treatments.^53–55^ However, the use of IL-17i in SpA patients has shown an increased incidence of IBD, or an exacerbation of pre-existing IBD.^56^ Two studies using IL-17i have been stopped early due to worsening of IBD symptoms.^57,58^ These surprising results are due to dysregulation of IL-23/IL-17 axis in IBD, since preclinical studies suggest a role of both IL-17 and IL-23 in pathogenesis of IBD. However, inhibition of these cytokines led to divergent effects in clinical trials.

It seems that IL-17 may have pathogenic and protective role in the gut epithelial barrier, predisposing to infection and inflammation, which can exacerbate or trigger IBD.^57^ As far as IL-17i safety is concerned, AEs were noted during the randomised clinical trials, which were quite similar to those of TNFαi. The commonest AEs were nasopharyngeal infections, local and fungal infections but no increase in the incidence of severe AEs was noted as compared to placebo. However, long-term observational studies regarding the role of IL-17i on radiological damage progression and drug discontinuation, are still missing.

### IL-23 inhibitors

IL-23 is a cytokine which plays a pivotal role in conjunction with IL-17 in the pathogenesis of SpA, referred to as the IL-17/IL-23 axis. IL-23 comprises two subunits p40 and p19, which when combined with IL-23R on target cells, trigger the activation of JAK pathway signaling, resulting in transcription of several cytokines like IL-17, IL-22 and TNFαi.^59^ RSK is an IL-23p19 inhibitor, UST is an IL-12/IL-23 p40 inhibitor, GUS is an IL-23 p19 inhibitor. All these molecules showed excellent results on the treatment of psoriasis^60–62^ and PsA.^63–65^ However, despite the above promising results of IL-23i in PsA and psoriasis, a phase III trial failed to demonstrate efficacy in patients with ax-SpA.^66,67^ The above divergent effects of IL-23 is difficult to understand. IL-23 is a member of IL-12 super family and is produced mostly by dendritic cells, monocytes and macrophages. IL-23 can stimulate Th17 cells to produce IL-17. However, there is evidence suggesting that tissue resident human entheseal γδT-cells, ILC-3, and MAIT can produce IL-17 independently of IL-23R transcript expression.^68^ Thus, differential activity of these cells in different tissues may contribute to IL-17 predominance versus IL-23 in driving inflammation and pathology in axSpA. It seems that IL-17 and IL-23, as well as TNFαi may enjoy distinct hierarchical roles in SpA spectrum (**Table 1**). On the contrary, in Crohn’s disease, UST and RSK inhibitors have demonstrated clinical efficacy in phase II and III trials.^69,70^ Regarding IL-23 inhibitors in uveitis, the results are not robust and data is missing. During the treatment with IL-12/IL-23i AEs were reported in patients with SpA which were quite similar to those of other bDMARDs. Among them, injection site reactions, respiratory infections, viral, and other opportunistic infections. Furthermore, long-term observational studies concerning its effects on radiological damage progression are still awaited.

### JAK inhibitors

In contrast to cytokine inhibitors, tsDMARDs are orally available, low molecular mass agents targeting multiple cytokines involved in SpA pathogenesis. More specifically, JAKi enter into cellular cytoplasm and direct intracellular signaling by inhibition of JAK and signal transducer and activation of transcription (STAT) pathway, and downstream gene expression.^71^ It seems that JAKi have a more robust effect by inhibiting several cytokines, such as IL-17, TNFα, IL-23 which are involved in SpA pathogenesis. Furthermore, anti-cytokine treatment can potentially induce immunogenicity leading to loss of efficacy and requires parenteral administration, in contrast to JAKi which are administered orally. Thus, a rational treatment option for SpA treatment is the use of JAKi. In this setting three JAKi are available for SpA. Tofacitinib (TOFA), which inhibits JAK1 and JAK3, upadacitinib (UPA) and filgotinib (FIL) which inhibit JAK1 and to a lesser extent other JAK molecules.^72^ UPA has been studied in AS patients in the SELECT-AXIS-1 and its extension study showing sustained efficacy over one year versus placebo and was not inferior to its comparator ADA.^73,74^ Furthermore, the SELECT-AXIS-2 evaluated UPA in nr-ax-SpA. It was found that UPA improved signs and symptoms in these patients.^75^ The SELECT-PsA-1 and its extension study assessed the use of UPA in PsA with inadequate response to bDMARDs. It was demonstrated that UPA efficacy was maintained over 56 months in these patients.^76–79^ In another study, UPA has been assessed in patients with Crohn’s disease. It has been shown that UPA induced endoscopic remission in a significant number of patients.^80^

TOFA in AS randomised clinical trials has demonstrated that it was superior to placebo in reducing signs and symptoms of the disease^81,82^ and showed a reduction of spinal inflammation on MRI.^83^ In addition, TOFA has been assessed in PsA patients with an inadequate response to non-biological agents. It was shown that TOFA was superior to placebo.^84^ In a subsequent study, TOFA has been evaluated in PsA patients with an inadequate response to TNFαi. It was found that TOFA was more effective than placebo in reducing disease activity in PsA patients.^85^

Finally, two randomised clinical trials using FIL, one in AS and the other in PsA, showed that the effectiveness of FIL was superior in AS and PsA vs placebo.^86,87^

Regarding JAKi safety, the most frequently reported AEs are infections which are quite similar to other bDMARDs.^88,89^ However, the most characteristic adverse effect of JAKi is the Herpes Zoster (HZ) infection or its reactivation.^90^ Other AEs include deep venous thrombosis (DVT), major adverse cardiovascular events (MACE), as well as malignancies. It was shown that JAKi were associated with higher incidence of malignancies compared to TNFαi.^91–93^ High risk of MACE and malignancies was confined to patients aged ≥65 years or smoking. These differentiating risk factors account for the excess risk observed in JAKi versus TNFαi.^94,95^ Thus, a minute and careful clinical evaluation and screening should be preferred for factors, such as chronic infections, thromboembolic events, as well as malignancies and cardiovascular disease before initiation of JAKi treatment. Finally, long-term observational studies are required to assess their sustained efficacy, safety and their effects on radiological damage progression.

## DISCUSSION

Our understanding regarding the pathogenesis of SpA has been expanded during the last two decades, which involves the discovery and the pivotal role and network of TNFα and the cytokines of the IL-17/IL-23 axis.^9–11^ This knowledge led to the introduction of biological therapies targeting several cytokines and molecules of the signaling JAK/STAT pathway. Nowadays, for the treatment of SpA physicians have in their armamentarium a plethora of biological therapies like TNFαi, IL-17i, IL-12/IL-23i as well as JAKi.^19–22^ On the other hand, it is important to emphasise that SpA is a heterogeneous disease with many clinical phenotypes and the cytokines mentioned above, are not equally expressed as their distribution and hierarchy differ in tissues and organs involved in SpA.^12,13^ In **Table 1**, the cytokine expression and its hierarchy in these patients is depicted. Having in mind this table, the question which arises is, which of the above, cytokine inhibitors or JAKi are capable of covering the entire spectrum of SpA phenotype? It is evident that TNFα is expressed with different hierarchy in all tissues and organs involved in SpA. On the other hand, IL-17 is lacking from the gut epithelium and IL-12/IL-23 is not expressed in the axial skeleton. Thus, to answer the above question, it seems that TNFαi along with JAKi is the most appropriate treatment for SpA. More specifically, IL-12/IL-23i are effective to treat psoriasis, PsA and IBD, but are ineffective in ax-SpA patients. In addition, long-term observational studies about their effectiveness in uveitis are still missing. Regarding the IL-17i, they seem very effective in patients with ax-SpA, psoriasis and PsA, but the results on concomitant IBD are not satisfactory. Furthermore, data regarding the effectiveness of IL-17i in uveitis and long-term observational studies on its safety is missing.^96^ Finally, JAKi which interfere with several cytokine signaling through the JAK/STAT pathway are very promising agents. These are low molecular mass drugs, administered orally, with clinical efficacy similar to TNFαi, covering almost the entire spectrum of SpA phenotypes except uveitis. Long term data on their efficacy in SpA is still waiting, but the most important concern of JAKi administration is the AEs, especially HZ, thromboembolic events, MACE and malignancies.^90–95^ The above AEs have been reported mostly in RA patients treated with JAKi and were associated with smoking, and patients >65 years old with cardiovascular comorbidities.^94^ However, patients with SpA, especially AS patients are young without significant comorbidities, while psoriasis, PsA and IBD could affect older patients, as well.

## CONCLUSIONS

TNFαi have shown a very good clinical profile in randomised long-term observational studies with an acceptable toxicity profile and cover the entire spectrum of SpA phenotype. TNFαi could also be administered in elderly patients with caution and after the appropriate screening. The efficacy of IL-17i was confirmed in many clinical trials in ax-SpA, psoriasis and PsA but the results on IBD and uveitis are not satisfactory, and in some cases, may ignite an IBD flare. As far as IL-12/IL-23i are concerned, these agents are very effective in psoriasis, IBD and PsA but failed to show relevant effectiveness in ax-SpA. Finally, JAKi are promising agents to treat almost the entire spectrum of SpA phenotype, but attention is required when these patients are administered in elderly patients, smokers, and patients with cardiovascular comorbidities. Therefore, JAKi should be preferred to treat young SpA patients without comorbidities, while TNFαi could be administered also in elderly patients with caution. In any case, the treatment of choice in SpA patients is individualised according to the clinical phenotype and after shared decision between patients and physicians.

## References

[1] Sieper J Poddubnyy D. Axial spondyloarthritis. Lancet 2017, 390(10089):73–84. 28110981 10.1016/S0140-6736(16)31591-4

[2] Navarro-Compán V Sepriano A El-Zorkany B van der Heijde D. Axial spondyloarthritis. Ann Rheum Dis 2021;80(12):1511–21. 34615639 10.1136/annrheumdis-2021-221035

[3] Drosos AA Venetsanopoulou AI Voulgari PV. Axial Spondyloarthritis: Evolving concepts regarding the disease's diagnosis and treatment. Eur J Intern Med 2023;S0953-6205(23)00221-2. doi: 10.1016/j.ejim.2023.06.026. 37414646

[4] Weber U Lambert RG Østergaard M Hodler J Pedersen SJ Maksymowych WP. The diagnostic utility of magnetic resonance imaging in spondylarthritis: an international multicenter evaluation of one hundred eighty-seven subjects. Arthritis Rheum 2010, 62(10):3048–58. 20496416 10.1002/art.27571

[5] Rudwaleit M Landewé R van der Heijde D Listing J Brandt J Braun J The development of Assessment of SpondyloArthritis international Society classification criteria for axial spondyloarthritis (part I): classification of paper patients by expert opinion including uncertainty appraisal. Ann Rheum Dis 2009;68(6):770–6. 19297345 10.1136/ard.2009.108217

[6] Rudwaleit M van der Heijde D Landewé R Listing J Akkoc N Brandt J The development of Assessment of SpondyloArthritis international Society classification criteria for axial spondyloarthritis (part II): validation and final selection. Ann Rheum Dis 2009;68(6):777–83. 19297344 10.1136/ard.2009.108233

[7] Chatzikyriakidou A Voulgari PV Drosos AA. What is the role of HLA-B27 in spondyloarthropathies? Autoimmun Rev 2011;10(8):464–8. 21296192 10.1016/j.autrev.2011.01.011

[8] Chatzikyriakidou A Voulgari PV Drosos AA. Non-HLA genes in ankylosing spondylitis: what meta-analyses have shown? Clin Exp Rheumatol 2014;32(5):735–9. 25068597

[9] Schett G Lories RJ D'Agostino MA Elewaut D Kirkham B Soriano ER Enthesitis: from pathophysiology to treatment. Nat Rev Rheumatol 2017;13(12):731–41. 29158573 10.1038/nrrheum.2017.188

[10] Sharip A Kunz J. Understanding the Pathogenesis of Spondyloarthritis. Biomolecules 2020;10(10):1461. 33092023 10.3390/biom10101461 PMC7588965

[11] Mauro D Thomas R Guggino G Lories R Brown MA Ciccia F. Ankylosing spondylitis: an autoimmune or autoinflammatory disease? Nat Rev Rheumatol 2021;17(7):387–404. 34113018 10.1038/s41584-021-00625-y

[12] Schett G McInnes IB Neurath MF. Reframing Immune-Mediated Inflammatory Diseases through Signature Cytokine Hubs. N Engl J Med 2021;385(7):628–39. 34379924 10.1056/NEJMra1909094

[13] Siebert S Millar NL McInnes IB. Why did IL-23p19 inhibition fail in AS: a tale of tissues, trials or translation? Ann Rheum Dis 2019;78(8):1015–8. 30297330 10.1136/annrheumdis-2018-213654 PMC6691857

[14] Elliott MJ Maini RN Feldmann M Kalden JR Antoni C Smolen JS Randomised double-blind comparison of chimeric monoclonal antibody to tumour necrosis factor alpha (cA2) versus placebo in rheumatoid arthritis. Lancet 1994;344(8930):1105–10. 7934491 10.1016/s0140-6736(94)90628-9

[15] Gorman JD Sack KE Davis JC Jr. Treatment of ankylosing spondylitis by inhibition of tumor necrosis factor alpha. N Engl J Med 2002;346(18):1349–56. 11986408 10.1056/NEJMoa012664

[16] Braun J Brandt J Listing J Zink A Alten R Golder W Treatment of active ankylosing spondylitis with infliximab: a randomised controlled multicentre trial. Lancet 2002;359(9313):1187–93. 11955536 10.1016/s0140-6736(02)08215-6

[17] Temekonidis TI Alamanos Y Nikas SN Bougias DV Georgiadis AN Voulgari PV Infliximab therapy in patients with ankylosing spondylitis: an open label 12 month study. Ann Rheum Dis 2003;62(12):1218–20. 14644863 10.1136/ard.2003.014258 PMC1754384

[18] Cardelli C Monti S Terenzi R Carli L. One year in review 2021: axial spondyloarthritis. Clin Exp Rheumatol 2021;39(6):1272–81. 34842133 10.55563/clinexprheumatol/jlyd1l

[19] Fragoulis GE Siebert S. Treatment strategies in axial spondyloarthritis: what, when and how? Rheumatology (Oxford) 2020;59(Suppl4):iv79–iv89. 33053192 10.1093/rheumatology/keaa435 PMC7566463

[20] Harrison SR Marzo-Ortega H. Have Therapeutics Enhanced Our Knowledge of Axial Spondyloarthritis? Curr Rheumatol Rep 2023, 25(3):56–67. 36652160 10.1007/s11926-023-01097-7 PMC9958165

[21] Ramiro S Nikiphorou E Sepriano A Ortolan A Webers C Baraliakos X ASAS-EULAR recommendations for the management of axial spondyloarthritis: 2022 update. Ann Rheum Dis 2023, 82(1):19–34. 36270658 10.1136/ard-2022-223296

[22] Coates LC Soriano ER Corp N Bertheussen H Callis Duffin K Campanholo CB GRAPPA Treatment Recommendations domain subcommittees. Group for Research and Assessment of Psoriasis and Psoriatic Arthritis (GRAPPA): updated treatment recommendations for psoriatic arthritis 2021. Nat Rev Rheumatol 2022;18(8):465–79. 35761070 10.1038/s41584-022-00798-0 PMC9244095

[23] Saougou I Markatseli TE Voulgari PV Drosos AA. Maintained clinical response of infliximab treatment in ankylosing spondylitis: a 6-year long-term study. Joint Bone Spine 2010;77(4):325–9. 20452801 10.1016/j.jbspin.2010.02.014

[24] Saougou I Markatseli TE Papagoras C Voulgari PV Alamanos Y Drosos AA. Sustained clinical response in psoriatic arthritis patients treated with anti-TNF agents: a 5-year open-label observational cohort study. Semin Arthritis Rheum 2011;40(5):398–406. 20843542 10.1016/j.semarthrit.2010.07.004

[25] Flouri ID Markatseli TE Boki KA Papadopoulos I Skopouli FN Voulgari PV Comparative Analysis and Predictors of 10-year Tumor Necrosis Factor Inhibitors Drug Survival in Patients with Spondyloarthritis: First-year Response Predicts Longterm Drug Persistence. J Rheumatol 2018;45(6):785–94. 29606666 10.3899/jrheum.170477

[26] Maksymowych WP Morency N Conner-Spady B Lambert RG. Suppression of inflammation and effects on new bone formation in ankylosing spondylitis: evidence for a window of opportunity in disease modification. Ann Rheum Dis 2013, 72(1):23–8. 22562977 10.1136/annrheumdis-2011-200859

[27] Lawson DO Eraso M Mbuagbaw L Joanes M Aves T Leenus A Tumor Necrosis Factor Inhibitor Dose Reduction for Axial Spondyloarthritis: A Systematic Review and Meta-Analysis of Randomized Controlled Trials. Arthritis Care Res (Hoboken) 2021;73(6):861–72. 32166872 10.1002/acr.24184

[28] Torgutalp M Rios Rodriguez V Dilbaryan A Proft F Protopopov M Verba M Treatment with tumour necrosis factor inhibitors is associated with a time-shifted retardation of radiographic spinal progression in patients with axial spondyloarthritis. Ann Rheum Dis 2022;81(9):1252–9. 35697486 10.1136/annrheumdis-2022-222324 PMC9380506

[29] Torgutalp M Rios Rodriguez V Proft F Protopopov M Verba M Rademacher J Treatment With Tumor Necrosis Factor Inhibitors Is Associated With a Time-Shifted Retardation of Radiographic Sacroiliitis Progression in Patients With Axial Spondyloarthritis: 10-Year Results From the German Spondyloarthritis Inception Cohort. Arthritis Rheumatol 2022;74(9):1515–23. 35437900 10.1002/art.42144

[30] Bruckmann NM Rischpler C Tsiami S Kirchner J Abrar DB Bartel T Effects of Anti-Tumor Necrosis Factor Therapy on Osteoblastic Activity at Sites of Inflammatory and Structural Lesions in Radiographic Axial Spondyloarthritis: A Prospective Proof-of-Concept Study Using Positron Emission Tomography/Magnetic Resonance Imaging of the Sacroiliac Joints and Spine. Arthritis Rheumatol 2022;74(9):1497–505. 35474641 10.1002/art.42149

[31] Ben-Shabat N Shabat A Watad A Kridin K Bragazzi NL McGonagle D Mortality in Ankylosing Spondylitis According to Treatment: A Nationwide Retrospective Cohort Study of 5,900 Patients From Israel. Arthritis Care Res (Hoboken) 2022;74(10):1614–22. 33973404 10.1002/acr.24616

[32] Marzo-Ortega H McGonagle D O'Connor P Emery P. Efficacy of etanercept for treatment of Crohn's related spondyloarthritis but not colitis. Ann Rheum Dis 2003;62(1):74–6. 12480676 10.1136/ard.62.1.74 PMC1754306

[33] Cholapranee A Hazlewood GS Kaplan GG Peyrin-Biroulet L Ananthakrishnan AN. Systematic review with meta-analysis: comparative efficacy of biologics for induction and maintenance of mucosal healing in Crohn's disease and ulcerative colitis controlled trials. Aliment Pharmacol Ther 2017;45(10):1291–302. 28326566 10.1111/apt.14030 PMC5395316

[34] Fragoulis GE Liava C Daoussis D Akriviadis E Garyfallos A Dimitroulas T. Inflammatory bowel diseases and spondyloarthropathies: From pathogenesis to treatment. World J Gastroenterol 2019;25(18):2162–76. 31143068 10.3748/wjg.v25.i18.2162 PMC6526158

[35] Lie E Lindström U Zverkova-Sandström T Olsen IC Forsbladd'Elia H Askling J Tumour necrosis factor inhibitor treatment and occurrence of anterior uveitis in ankylosing spondylitis: results from the Swedish biologics register. Ann Rheum Dis 2017;76(9):1515–21. 28254789 10.1136/annrheumdis-2016-210931

[36] van Bentum RE Heslinga SC Nurmohamed MT Gerards AH Griep EN Koehorst CBJM Reduced Occurrence Rate of Acute Anterior Uveitis in Ankylosing Spondylitis treated with Golimumab - The GO-EASY Study. J Rheumatol 2019;46(2):153–59. 30385705 10.3899/jrheum.180312

[37] Venetsanopoulou AI Mavridou K Voulgari PV Drosos AA. Cutaneous immune-related phenomena in patients with inflammatory arthritides treated with biological therapies: Clinical and pathophysiological considerations. Semin Arthritis Rheum 2023;63:152272. doi: 10.1016/j.semarthrit.2023.152272. 37788595

[38] Drosos AA Pelechas E Kaltsonoudis E Markatseli TE Voulgari PV. Biologic Therapies and Autoimmune Phenomena. Mediterr J Rheumatol 2021;32(2):96–103. 34447904 10.31138/mjr.32.2.96 PMC8369271

[39] Kaltsonoudis E Zikou AK Voulgari PV Konitsiotis S Argyropoulou MI Drosos AA. Neurological adverse events in patients receiving anti-TNF therapy: a prospective imaging and electrophysiological study. Arthritis Res Ther 2014;16(3):R125. 24938855 10.1186/ar4582 PMC4229940

[40] Pelechas E Memi T Markatseli TE Voulgari PV Drosos AA. Adalimumab-induced myasthenia gravis: case-based review. Rheumatol Int 2020;40(11):1891–4. 32322981 10.1007/s00296-020-04587-4

[41] Park W Yoo DH Miranda P Brzosko M Wiland P Gutierrez-Ureña S Efficacy and safety of switching from reference infliximab to CT-P13 compared with maintenance of CT-P13 in ankylosing spondylitis: 102-week data from the PLANETAS extension study. Ann Rheum Dis 2017;76(2):346–54. 27117698 10.1136/annrheumdis-2015-208783 PMC5284340

[42] Kaltsonoudis E Pelechas E Voulgari PV Drosos AA. Maintained Clinical Remission in Ankylosing Spondylitis Patients Switched from Reference Infliximab to Its Biosimilar: An 18-Month Comparative Open-Label Study. J Clin Med 2019;8(7):956. 31269678 10.3390/jcm8070956 PMC6679061

[43] McGeachy MJ Cua DJ Gaffen SL. The IL-17 Family of Cytokines in Health and Disease. Immunity 2019;50(4):892–906. 30995505 10.1016/j.immuni.2019.03.021 PMC6474359

[44] Rosine N Miceli-Richard C. Innate Cells: The Alternative Source of IL-17 in Axial and Peripheral Spondyloarthritis? Front Immunol 2021;11:553742. 33488572 10.3389/fimmu.2020.553742 PMC7821711

[45] Jandus C Bioley G Rivals JP Dudler J Speiser D Romero P. Increased numbers of circulating polyfunctional Th17 memory cells in patients with seronegative spondylarthritides. Arthritis Rheum 2008;58(8):2307–17. 18668556 10.1002/art.23655

[46] Pavelka K Kivitz A Dokoupilova E Blanco R Maradiaga M Tahir H Efficacy, safety, and tolerability of secukinumab in patients with active ankylosing spondylitis: a randomized, double-blind phase 3 study, MEASURE 3. Arthritis Res Ther 2017;19(1):285. 29273067 10.1186/s13075-017-1490-y PMC5741872

[47] Kivitz AJ Wagner U Dokoupilova E Supronik J Martin R Talloczy Z Efficacy and Safety of Secukinumab 150 mg with and Without Loading Regimen in Ankylosing Spondylitis: 104-week Results from MEASURE 4 Study. Rheumatol Ther 2018;5(2):447–62. 30121827 10.1007/s40744-018-0123-5 PMC6251842

[48] Behrens F Sewerin P de Miguel E Patel Y Batalov A Dokoupilova E ACHILLES study group. Efficacy and safety of secukinumab in patients with spondyloarthritis and enthesitis at the Achilles tendon: results from a phase 3b trial. Rheumatology (Oxford) 2022;61(7):2856–66. 34730795 10.1093/rheumatology/keab784 PMC9258542

[49] Deodhar A Blanco R Dokoupilová E Hall S Kameda H Kivitz AJ Improvement of Signs and Symptoms of Nonradiographic Axial Spondyloarthritis in Patients Treated With Secukinumab: Primary Results of a Randomized, Placebo-Controlled Phase III Study. Arthritis Rheumatol 2021;73(1):110–20. 32770640 10.1002/art.41477 PMC7839589

[50] van der Heijde D Cheng-Chung Wei J Dougados M Mease P Deodhar A Maksymowych COAST-V study group. Ixekizumab, an interleukin-17A antagonist in the treatment of ankylosing spondylitis or radiographic axial spondyloarthritis in patients previously untreated with biological disease-modifying anti-rheumatic drugs (COAST-V): 16 week results of a phase 3 randomised, double-blind, active-controlled and placebo-controlled trial. Lancet 2018;392(10163):2441–51. 30360964 10.1016/S0140-6736(18)31946-9

[51] Deodhar A Poddubnyy D Pacheco-Tena C Salvarani C Lespessailles E Rahman P COAST-W Study Group. Efficacy and Safety of Ixekizumab in the Treatment of Radiographic Axial Spondyloarthritis: Sixteen-Week Results From a Phase III Randomized, Double-Blind, Placebo-Controlled Trial in Patients With Prior Inadequate Response to or Intolerance of Tumor Necrosis Factor Inhibitors. Arthritis Rheumatol 2019;71(4):599–611. 30343531 10.1002/art.40753 PMC6593790

[52] Deodhar A van der Heijde D Gensler LS Kim TH Maksymowych WP Østergaard M COAST-X Study Group. Ixekizumab for patients with non-radiographic axial spondyloarthritis (COAST-X): a randomised, placebo-controlled trial. Lancet. 2020, 395(10217):53–64. 31813637 10.1016/S0140-6736(19)32971-X

[53] Pina Vegas L Penso L Claudepierre P Sbidian E. Long-term Persistence of First-line Biologics for Patients With Psoriasis and Psoriatic Arthritis in the French Health Insurance Database. JAMA Dermatol 2022;158(5):513–522. 35319735 10.1001/jamadermatol.2022.0364 PMC8943623

[54] Gossec L Baraliakos X Kerschbaumer A de Wit M McInnes I Dougados M EULAR recommendations for the management of psoriatic arthritis with pharmacological therapies: 2019 update. Ann Rheum Dis 2020;79(6):700–12. 32434812 10.1136/annrheumdis-2020-217159 PMC7286048

[55] Singh JA Guyatt G Ogdie A Gladman DD Deal C Deodhar A Special Article: 2018 American College of Rheumatology/National Psoriasis Foundation Guideline for the Treatment of Psoriatic Arthritis. Arthritis Rheumatol 2019;71(1):5–32. 30499246 10.1002/art.40726 PMC8218333

[56] Fauny M Moulin D D'Amico F Netter P Petitpain N Arnone D Paradoxical gastrointestinal effects of interleukin-17 blockers. Ann Rheum Dis 2020;79(9):1132–8. 32719044 10.1136/annrheumdis-2020-217927

[57] Hueber W Sands BE Lewitzky S Vandemeulebroecke M Reinisch W Higgins PD Secukinumab in Crohn's Disease Study Group. Secukinumab, a human anti-IL-17A monoclonal antibody, for moderate to severe Crohn's disease: unexpected results of a randomised, double-blind placebo-controlled trial. Gut 2012;61(12):1693–700. 22595313 10.1136/gutjnl-2011-301668 PMC4902107

[58] Targan SR Feagan B Vermeire S Panaccione R Melmed GY Landers C A Randomized, Double-Blind, Placebo-Controlled Phase 2 Study of Brodalumab Patients With Moderate-to-Severe Crohn's Disease. Am J Gastroenterol 2016;111(11):1599–607. 27481309 10.1038/ajg.2016.298

[59] Schinocca C Rizzo C Fasano S Grasso G La Barbera L Ciccia F Role of the IL-23/IL-17 Pathway in Rheumatic Diseases: An Overview. Front Immunol 2021;12:637829. 33692806 10.3389/fimmu.2021.637829 PMC7937623

[60] Leonardi CL Kimball AB Papp KA Yeilding N Guzzo C Wang Y PHOENIX 1 study investigators. Efficacy and safety of ustekinumab, a human interleukin-12/23 monoclonal antibody, in patients with psoriasis: 76-week results from a randomised, double-blind, placebo-controlled trial (PHOENIX 1). Lancet 2008;371(9625):1665–74. 18486739 10.1016/S0140-6736(08)60725-4

[61] Papp KA Langley RG Lebwohl M Krueger GG Szapary P Yeilding N PHOENIX 2 study investigators. Efficacy and safety of ustekinumab, a human interleukin-12/23 monoclonal antibody, in patients with psoriasis: 52-week results from a randomised, double-blind, placebo-controlled trial (PHOENIX 2). Lancet 2008;371(9625):1675–84. 18486740 10.1016/S0140-6736(08)60726-6

[62] Gordon KB Strober B Lebwohl M Augustin M Blauvelt A Poulin Y Efficacy and safety of risankizumab in moderate-to-severe plaque psoriasis (UltIMMa-1 and UltIMMa-2): results from two double-blind, randomised, placebo-controlled and ustekinumab-controlled phase 3 trials. Lancet 2018;392(10148):650–61. 30097359 10.1016/S0140-6736(18)31713-6

[63] Deodhar A Helliwell PS Boehncke WH Kollmeier AP Hsia EC Subramanian RA DISCOVER-1 Study Group. Guselkumab in patients with active psoriatic arthritis who were biologic-naive or had previously received TNFα inhibitor treatment (DISCOVER-1): a double-blind, randomised, placebo-controlled phase 3 trial. Lancet 2020;395(10230):1115–25. 32178765 10.1016/S0140-6736(20)30265-8

[64] Mease PJ Rahman P Gottlieb AB Kollmeier AP Hsia EC Xu XL DISCOVER-2 Study Group. Guselkumab in biologic-naive patients with active psoriatic arthritis (DISCOVER-2): a double-blind, randomised, placebo-controlled phase 3 trial. Lancet 2020;395(10230):1126–36. 32178766 10.1016/S0140-6736(20)30263-4

[65] Coates LC Gossec L Theander E Bergmans P Neuhold M Karyekar CS Efficacy and safety of guselkumab in patients with active psoriatic arthritis who are inadequate responders to tumour necrosis factor inhibitors: results through one year of a phase IIIb, randomised, controlled study (COSMOS). Ann Rheum Dis 2022;81(3):359–69. 34819273 10.1136/annrheumdis-2021-220991 PMC8862038

[66] Baeten D Østergaard M Wei JC Sieper J Järvinen P Tam LS Risankizumab, an IL-23 inhibitor, for ankylosing spondylitis: results of a randomised, double-blind, placebo-controlled, proof-of-concept, dose-finding phase 2 study. Ann Rheum Dis 2018;77(9):1295–302. 29945918 10.1136/annrheumdis-2018-213328 PMC6104676

[67] Deodhar A Gensler LS Sieper J Clark M Calderon C Wang Y Three Multicenter, Randomized, Double-Blind, Placebo-Controlled Studies Evaluating the Efficacy and Safety of Ustekinumab in Axial Spondyloarthritis. Arthritis Rheumatol 2019;71(2):258–70. 30225992 10.1002/art.40728

[68] Lee JS Tato CM Joyce-Shaikh B Gulen MF Cayatte C Chen Y Interleukin-23-Independent IL-17 Production Regulates Intestinal Epithelial Permeability. Immunity 2015;43(4):727–38. 26431948 10.1016/j.immuni.2015.09.003 PMC6044435

[69] Feagan BG Sandborn WJ Gasink C Jacobstein D Lang Y Friedman JR UNITI–IM-UNITI Study Group Ustekinumab as Induction and Maintenance Therapy for Crohn's Disease. N Engl J Med 2016;375(20):1946–60. 27959607 10.1056/NEJMoa1602773

[70] Feagan BG Sandborn WJ D'Haens G Panés J Kaser A Ferrante M Induction therapy with the selective interleukin-23 inhibitor risankizumab in patients with moderate-to-severe Crohn's disease: a randomised, double-blind, placebo-controlled phase 2 study. Lancet 2017;389(10080):1699–709. 28411872 10.1016/S0140-6736(17)30570-6

[71] McInnes IB Szekanecz Z McGonagle D Maksymowych WP Pfeil A Lippe R A review of JAK-STAT signalling in the pathogenesis of spondyloarthritis and the role of JAK inhibition. Rheumatology (Oxford) 2022;61(5):1783–794. 34668515 10.1093/rheumatology/keab740 PMC9071532

[72] Tanaka Y Luo Y O'Shea JJ Nakayamada S. Janus kinase-targeting therapies in rheumatology: a mechanisms-based approach. Nat Rev Rheumatol 2022;18(3):133–45. 34987201 10.1038/s41584-021-00726-8 PMC8730299

[73] van der Heijde D Song IH Pangan AL Deodhar A van den Bosch F Maksymowych WP Efficacy and safety of upadacitinib in patients with active ankylosing spondylitis (SELECT-AXIS 1): a multicentre, randomised, double-blind, placebo-controlled, phase 2/3 trial. Lancet 2019;394(10214):2108–17. 31732180 10.1016/S0140-6736(19)32534-6

[74] Deodhar A van der Heijde D Sieper J Van den Bosch F Maksymowych WP Kim TH Safety and Efficacy of Upadacitinib in Patients With Active Ankylosing Spondylitis and an Inadequate Response to Nonsteroidal Antiinflammatory Drug Therapy: One-Year Results of a Double-Blind, Placebo-Controlled Study and Open-Label Extension. Arthritis Rheumatol 2022;74(1):70–80. 34196498 10.1002/art.41911 PMC9299108

[75] Deodhar A Van den Bosch F Poddubnyy D Maksymowych WP van der Heijde D Kim TH Upadacitinib for the treatment of active non-radiographic axial spondyloarthritis (SELECT-AXIS 2): a randomised, double-blind, placebo-controlled, phase 3 trial. Lancet 2022;400(10349):369–79. 35908570 10.1016/S0140-6736(22)01212-0

[76] McInnes IB Anderson JK Magrey M Merola JF Liu Y Kishimoto M Trial of Upadacitinib and Adalimumab for Psoriatic Arthritis. N Engl J Med 2021;384(13):1227–39. 33789011 10.1056/NEJMoa2022516

[77] McInnes IB Kato K Magrey M Merola JF Kishimoto M Pacheco-Tena C Upadacitinib in patients with psoriatic arthritis and an inadequate response to non-biological therapy: 56-week data from the phase 3 SELECT-PsA 1 study. RMD Open 2021;7(3):e001838. 34663636 10.1136/rmdopen-2021-001838 PMC8524381

[78] Mease PJ Lertratanakul A Anderson JK Papp K Van den Bosch F Tsuji S Upadacitinib for psoriatic arthritis refractory to biologics: SELECT-PsA 2. Ann Rheum Dis 2021;80(3):312–20. 33272960 10.1136/annrheumdis-2020-218870 PMC7892371

[79] Mease PJ Lertratanakul A Papp KA van den Bosch FE Tsuji S Dokoupilova E Upadacitinib in Patients with Psoriatic Arthritis and Inadequate Response to Biologics: 56-Week Data from the Randomized Controlled Phase 3 SELECT-PsA 2 Study. Rheumatol Ther 2021;8(2):903–19. 33913086 10.1007/s40744-021-00305-z PMC8217417

[80] Sandborn WJ Feagan BG Loftus EV Jr Peyrin-Biroulet L Van Assche G D'Haens G Efficacy and Safety of Upadacitinib in a Randomized Trial of Patients With Crohn's Disease. Gastroenterology 2020;158(8):2123–2138.e8. 32044319 10.1053/j.gastro.2020.01.047

[81] van der Heijde D Deodhar A Wei JC Drescher E Fleishaker D Hendrikx T Tofacitinib in patients with ankylosing spondylitis: a phase II, 16-week, randomised, placebo-controlled, dose-ranging study. Ann Rheum Dis 2017;76(8):1340–7. 28130206 10.1136/annrheumdis-2016-210322 PMC5738601

[82] Deodhar A Sliwinska-Stanczyk P Xu H Baraliakos X Gensler LS Fleishaker D Tofacitinib for the treatment of ankylosing spondylitis: a phase III, randomised, double-blind, placebo-controlled study. Ann Rheum Dis 2021;80(8):1004–13. 33906853 10.1136/annrheumdis-2020-219601 PMC8292568

[83] Maksymowych WP van der Heijde D Baraliakos X Deodhar A Sherlock SP Li D Tofacitinib is associated with attainment of the minimally important reduction in axial magnetic resonance imaging inflammation in ankylosing spondylitis patients. Rheumatology (Oxford) 2018;57(8):1390–9. 29718421 10.1093/rheumatology/key104 PMC6055606

[84] Mease P Hall S FitzGerald O van der Heijde D Merola JF Avila-Zapata F Tofacitinib or Adalimumab versus Placebo for Psoriatic Arthritis. N Engl J Med 2017;377(16):1537–50. 29045212 10.1056/NEJMoa1615975

[85] Gladman D Rigby W Azevedo VF Behrens F Blanco R Kaszuba A Tofacitinib for Psoriatic Arthritis in Patients with an Inadequate Response to TNF Inhibitors. N Engl J Med 2017;377(16):1525–36. 29045207 10.1056/NEJMoa1615977

[86] Mease P Coates LC Helliwell PS Stanislavchuk M Rychlewska-Hanczewska A Dudek A Efficacy and safety of filgotinib, a selective Janus kinase 1 inhibitor, in patients with active psoriatic arthritis (EQUATOR): results from a randomised, placebo-controlled, phase 2 trial. Lancet 2018;392(10162):2367–77. 30360969 10.1016/S0140-6736(18)32483-8

[87] van der Heijde D Baraliakos X Gensler LS Maksymowych WP Tseluyko V Nadashkevich O Efficacy and safety of filgotinib, a selective Janus kinase 1 inhibitor, in patients with active ankylosing spondylitis (TORTUGA): results from a randomised, placebo-controlled, phase 2 trial. Lancet 2018;392(10162):2378–87. 30360970 10.1016/S0140-6736(18)32463-2

[88] Winthrop KL. The emerging safety profile of JAK inhibitors in rheumatic disease. Nat Rev Rheumatol 2017;13(4):234–43. 28250461 10.1038/nrrheum.2017.23

[89] Venetsanopoulou AI Voulgari PV Drosos AA. Janus kinase versus TNF inhibitors: where we stand today in rheumatoid arthritis. Expert Rev Clin Immunol 2022;18(5):485–93. 35535405 10.1080/1744666X.2022.2064275

[90] Redeker I Albrecht K Kekow J Burmester GR Braun J Schäfer M Risk of herpes zoster (shingles) in patients with rheumatoid arthritis under biologic, targeted synthetic and conventional synthetic DMARD treatment: data from the German RABBIT register. Ann Rheum Dis 2022;81(1):41–7. 34321218 10.1136/annrheumdis-2021-220651 PMC8762036

[91] Cohen SB. JAK inhibitors and VTE risk: how concerned should we be? Nat Rev Rheumatol 2021;17(3):133–4. 33452499 10.1038/s41584-021-00575-5

[92] Yates M Mootoo A Adas M Bechman K Rampes S Patel V Venous Thromboembolism Risk With JAK Inhibitors: A Meta-Analysis. Arthritis Rheumatol 2021;73(5):779–88. 33174384 10.1002/art.41580

[93] Curtis JR Yamaoka K Chen YH Bhatt DL Gunay LM Sugiyama N Malignancy risk with tofacitinib versus TNF inhibitors in rheumatoid arthritis: results from the open-label, randomised controlled ORAL Surveillance trial. Ann Rheum Dis 2023;82(3):331–43. 36600185 10.1136/ard-2022-222543 PMC9933177

[94] Kristensen LE Danese S Yndestad A Wang C Nagy E Modesto I Identification of two tofacitinib subpopulations with different relative risk versus TNF inhibitors: an analysis of the open label, randomised controlled study ORAL Surveillance. Ann Rheum Dis 2023;82(7):901–10. 36931693 10.1136/ard-2022-223715 PMC10314011

[95] Russell MD Stovin C Alveyn E Adeyemi O Chan CKD Patel V JAK inhibitors and the risk of malignancy: a meta-analysis across disease indications. Ann Rheum Dis 2023, 82(8):1059–67. 37247942 10.1136/ard-2023-224049 PMC10359573

[96] Webers C Ortolan A Sepriano A Falzon L Baraliakos X Landewé RBM Efficacy and safety of biological DMARDs: a systematic literature review informing the 2022 update of the ASAS-EULAR recommendations for the management of axial spondyloarthritis. Ann Rheum Dis 2023;82(1):130–41. 36270657 10.1136/ard-2022-223298

